# Diagnostic Accuracy of S100B Urinary Testing at Birth in Full-Term Asphyxiated Newborns to Predict Neonatal Death

**DOI:** 10.1371/journal.pone.0004298

**Published:** 2009-02-02

**Authors:** Diego Gazzolo, Alessandro Frigiola, Moataza Bashir, Iman Iskander, Hala Mufeed, Hanna Aboulgar, Pierluigi Venturini, Mauro Marras, Giovanni Serra, Rosanna Frulio, Fabrizio Michetti, Felice Petraglia, Raul Abella, Pasquale Florio

**Affiliations:** 1 Department of Obstetrics, Pediatrics and Neuroscience, G. Gaslini Children's Hospital University of Genoa, Genoa, Italy; 2 Department of Maternal Fetal and Neonatal Health G. Garibaldi Hospital, Catania, Italy; 3 Perinatal Research Laboratory, Department of Cardiac Surgery, S. Donato Milanese University Hospital, S. Donato Milanese, Italy; 4 Department of Neonatology, Cairo University, Cairo, Egypt; 5 Institute of Anatomy, Catholic University, Rome, Italy; 6 Department of Pediatrics, Obstetrics and Reproductive Medicine, University of Siena, Siena, Italy; JARING, Malaysia

## Abstract

**Background:**

Neonatal death in full-term infants who suffer from perinatal asphyxia (PA) is a major subject of investigation, since few tools exist to predict patients at risk of ominous outcome. We studied the possibility that urine S100B measurement may identify which PA-affected infants are at risk of early postnatal death.

**Methodology/Principal Findings:**

In a cross-sectional study between January 1, 2001 and December 1, 2006 we measured S100B protein in urine collected from term infants (n = 132), 60 of whom suffered PA. According to their outcome at 7 days, infants with PA were subsequently classified either as asphyxiated infants complicated by hypoxic ischemic encephalopathy with no ominous outcome (HIE Group; n = 48), or as newborns who died within the first post-natal week (Ominous Outcome Group; n = 12). Routine laboratory variables, cerebral ultrasound, neurological patterns and urine concentrations of S100B protein were determined at first urination and after 24, 48 and 96 hours. The severity of illness in the first 24 hours after birth was measured using the Score for Neonatal Acute Physiology-Perinatal Extension (SNAP-PE). Urine S100B levels were higher from the first urination in the ominous outcome group than in healthy or HIE Groups (p<0.001 for all), and progressively increased. Multiple logistic regression analysis showed a significant correlation between S100B concentrations and the occurrence of neonatal death. At a cut-off >1.0 µg/L S100B had a sensitivity/specificity of 100% for predicting neonatal death.

**Conclusions/Significance:**

Increased S100B protein urine levels in term newborns suffering PA seem to suggest a higher risk of neonatal death for these infants.

## Introduction

Neonatal death in full-term infants suffering perinatal asphyxia (PA) is a major subject of concern, since to date no clinical, biochemical or biophysical tools exist to predict which patients are at risk of ominous outcome [1]. Epidemiological studies have highlighted the relevance of the timing of the hypoxic insult, which in the majority of cases occurs in the pre-perinatal period [2]. To date, the possibility of detecting infants at risk of this severe complication is limited since clinical, laboratory and standard monitoring procedures may be silent or unreliable. A practical and sensitive marker able to offer neonatologists a useful tool for clinical and ethical purposes is therefore eagerly awaited.

In the last decade a brain constituent, S100B protein, has been proposed as a consolidated marker of brain damage [3], since elevated S100B concentrations in biological fluids have been found in brain-damaged adults, infants and fetuses [3]–[12]. S100B belongs to a multigenic family of calcium-modulated proteins (S100 proteins), mostly of low molecular weight (approximately 10,000 Da), first identified as a protein fraction detectable in brain (in glial and Schwann cells, in specific neuronal subpopulations) and named S100 because of its solubility in a 100% saturated solution of ammonium sulfate [4].

With regard to perinatal medicine, it is noteworthy that S100B has recently been shown to be a reliable diagnostic test for predicting newborns at risk of pre-perinatal death [13], [14]. Of the biological fluids in which this protein has been assessed, urine appears to be the most suitable, because it can be collected easily and sampling can be repeated without additional risks for the newborn.

The present study aimed to evaluate whether the measurement of S100B in urine may represent a useful tool to identify a risk of early postnatal death in full-term newborns affected by PA.

## Results

### Clinical and laboratory parameters

At birth, no significant differences regarding weight, gestational age and gender distribution were found between neonatal death and control groups (P>0.05 for all). Of the 12 infants with ominous outcome, 10 developed severe HIE whilst, in the group complicated by PA with no ominous outcome (HIE group), 36 out of 48 developed mild HIE and 12 out of 48 severe HIE. The incidence of ARDS was significantly different in the neonatal death group compared with controls (P<0.001). Clinical findings, neonatal outcomes and laboratory parameters of all the studied infants are shown in Tables 1 and 2. As expected, Apgar scores at birth, at the 1^st^ and 5^th^ minutes, pH, PvCO_2_, and base excess were significantly different in newborns who suffered PA (both HIE and ominous outcome groups) and in the healthy group (P<0.001 for all). However, no differences were found when the ominous outcome group was compared with the HIE group (P>0.05, for all) (Table 2), even at the 24, 48, and 96-hour time-points (P>0.05 for all) (data not shown).

**Table 1:** Maternal and neonatal characteristics at birth in infants without overt neurological syndrome [healthy group (n = 72)], in infants with asphyxia complicated by hypoxic ischemic encephalopathy with no ominous outcome (HIE Group; n = 48)] and in newborns affected by PA and with ominous outcome (Ominous Outcome Group).

	Healthy Group (n = 72)	HIE Group (n = 48)	Ominous outcome Group (n = 12)
Mean maternal age *(years)*	30	31	30
Cesarean Section *(yes/total)*	20/20*	18/18*	31/96
Gestational age *(wks)*	38.4±2.1	38.1±1.6	39.1±1.4
Sex: Male/Female *(n°)*	38/34	25/23	7/5
Birth weight *(g)*	3,100±507	3,198±414	3,451±231
Apgar 1^st^ min <7 *(yes/total)*	0/72	48/48	12/12
Apgar 5^th^ min <7 *(yes/total)*	0/72	48/48	12/12
RDS *(yes/total)*	0/72	7/48*	0/96
Mechanical Ventilation or NCPAP support *(yes/total)*	0/72	33/48*	12/12
HIE according to Sarnat's test *(yes/total)*			
• Moderate (yes/total)	0/72	36/48*	2/12
• Severe (yes/total)	0/72	12/48*	10/12
Cerebral US (normal/hyperechogenicity/bleeding)			
• Birth	Not performed	28/20/0	2/10/0
• 12 hours	Not performed	28/20/0	2/10/0
• 24 hours	Not performed	28/20/0	0/12/0
• 72 hours	72/0/0	28/20/0	0/12/0
Prechtl's Test (normal/suspect/abnormal)			
• Birth	0/0/0*	0/18/0*	0/4/4
• 24 hours	0/0/0*	0/6/3*	0/8/4
• 48 hours	0/0/0*	0/6/3*	0/6/6
• 96 hours	0/0/0*	0/6/3*	0/6/6
SNAP-PE score at NICU admission	32±4	36±9	40±9

Values are expressed as mean±SD.

* P<0.05 vs controls.

**Table 2:** Neonatal monitoring parameters at birth in infants without overt neurological syndrome [healthy (n = 72) and HIE (n = 48) Groups] and in newborns affected by PA and with ominous outcome (Ominous Outcome Group, n = 12).

	**Healthy Group (n = 72)**	**HIE Group (n = 48)**	**Ominous outcome Group (n = 12)**
RBC count *(10^6^/mm^3^)*	4.1±0.5	3.91±0.5	3.93±0.3
Hemoglobin *(g/dL)*	13.8±0.2	13.7±0.2	13.6±0.4
Hematocrit rate %	41.5±1.5	41.2±2.2	40.7±1.9
Venous blood pH	7.34±0.1	7.05±0.1*	7.00±0.1*
Partial venous CO_2_ pressure *(mmHg)*	42.4±4.1	78.6±9.2*	61.6±17.5*
Partial venous O_2_ pressure *(mmHg)*	41.2±4.9	17.1±7.6	31.6±23.4
Base excess	0.4±2.1	−12.3±2.2*	−14.1±5.1*
Na^+^ *(mmol/L)*	141±3	138±5	140±3
K^+^ *(mmol/L)*	4.1±0.3	4.7±0.3	4.1±0.2
Ca^++^ *(mmol/L)*	1.19±0.02	1.11±0.06	1.12±0.1
Plasma glucose *(mmol/L)*	4.2±0.2	4.2±1.1	4.1±1.2
Urea *(mg/dL)*	39.2±8.8	35.2±15.8	42.6±10.6
Creatinine *(mg/dL)*	0.81±0.3	0.87±0.28	0.93±0.11
Urine gravity	1,010±4.4	1,012±2.4	1,011±3.3

Values are expressed as mean±SD.

* P<0.05 vs healthy Group.

**Abbreviations:** RBC: Red Blood Cells

The mean (SD) SNAP-PE score recorded within the first 24 hours after birth was significantly lower in healthy controls (31.8±4.1) than in newborns with PA with no ominous outcome (HIE Group: 36.2±8.7), and in infants who died within the first week after birth (Ominous Outcome Group: 39.7±8.8) (P<0.05 for all), while not differing between the two groups with PA classified according to their outcome (data not shown).

### Neurodevelopmental outcome and cerebral ultrasound findings

Cerebral ultrasound scans performed 24 hours from birth were negative in the 72 uncomplicated control infants and in 20 out of 48 control infants with perinatal asphyxia. Echographic patterns suggestive of brain edema and/or periventricular hyperechogenicity were observed in 28 out of 48 control infants with perinatal asphyxia and in 10 out of 12 infants of the neonatal Ominous Outcome Group. None of the monitored infants showed congenital cerebral malformations or bleeding. Identical cerebral echographic patterns were observed at the 48 and 96-hr time-points, except for the remaining 2 infants of the neonatal death group, in which brain edema and/or periventricular hyperechogenicity were apparent.

Neurological examination at NICU admission was negative in the 72 control infants, whilst in 18 out of 48 controls with perinatal asphyxia it was suspect (hypo/hypertonia: n = 11; hyperexcitability: n = 7). Neurological patterns remained superimposable at the 24, 48 and 96-hr time-points. In the neonatal death group isolated hyperexcitability (n = 4) and hypo/hypertonia (n = 4) symptoms were recorded, and between the 24 and 96-hr time-points there was an impairment in neurological examination patterns, with hyperexcitability (n = 6) and hypo/hypertonia (n = 6).

In the neonatal death group 5 died within the first 120 hours from birth and the remaining 7 newborns died of cardiopulmonary failure within the first week.

### Urine S100B levels and the prediction of ominous outcome

**Figure 1:**
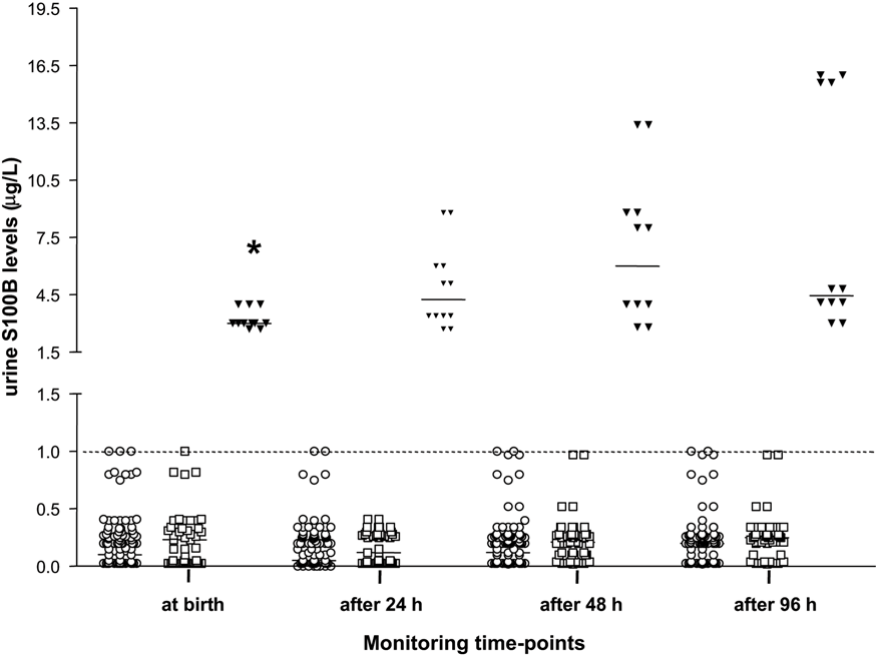
S100B levels in urine at first urination. S100B concentrations were significantly (p<0.001) higher in newborns who died within the first week of age (Ominous Outcome Group: black triangles) than in healthy controls (open circles), and in infants suffering PA without ominous outcome (HIE Group: open squares). ROC curve analysis shows that the S100B measurement as a diagnostic test has a sensitivity and a specificity of 100% at a cut-off of 1.0 µg/L (shown by the dotted line) with 100% positive predictive value and 100% negative predictive value. Horizontal bars indicate the median value for each group. *P<0.05 versus 24 h and <0.01 versus 96 h monitoring time-points.

Urine S100B protein levels were detectable in all the patients examined. While the levels did not change during monitoring in either healthy neonates or newborns affected by PA with a good outcome, in the group with ominous outcome they increased, peaking after 48 and 96 hrs (P = 0.027) (Figure 1). In these infants, S100B concentrations were significantly higher at all monitoring time-points (P<0.001 for all) than in both healthy and HIE infants (P<0.001 for all), whilst they did not differ between the latter two groups (Figure 1) (Table 3).

**Table 3:** Urine S100B concentrations (µg/L) expressed as median [lower and upper 95% Confidence Interval (CI)] at first urination (0), and at 24 (1), 48 (2) and 96 (3) hours in healthy Group (n = 72), in newborns affected by PA without overt neurologic syndrome (n = 48, HIE Group) and with ominous outcome (n = 12, Ominous Outcome Group).

S100B (µg/L)	Healthy Group (n = 72)			HIE Group (n = 48)			Ominous Outcome Group (n = 12)		
	Median	Lower CI_95%_	Upper CI_95%_	Median	Lower CI_95%_	Upper CI_95%_	Median	Lower CI_95%_	Upper CI_95%_
First urination (0)*	0.1	0.15	0.23	0.23	0.17	0.31	3.0	2.94	3.63
24 hours (1)*	0.05	0.12	0.19	0.12	0.12	0.2	4.25	3.52	6.27
48 hours (2)*	0.12	0.15	0.23	0.21	0.17	0.29	6.0	4.4	9.26
96 hours (3)*	0.2	0.16	0.24	0.25	0.2	0.31	4.45	4.22	11.64

Urinary S100B levels were significantly higher in the neonatal death group at all monitoring time-points (p<0.001, for all).

* p<0.001 vs controls

Multiple logistic regression analysis with neonatal death as dependent variable showed a positive significant correlation only between S100B blood levels (P<0.001) and the occurrence of neonatal death among a series of monitoring parameters (odds ratio at birth and at 48 hours from birth: 11.42 and 9.15, respectively).

On the basis of these results and the outcome after 7******days, newborns were grouped as normal (n = 120, comprising healthy newborns and HIE infants) and pathological (n = 12, infants with PA and ominous outcome). Table 4 shows the reliability/efficiency of S100B protein levels at different monitoring time-points in predicting the occurrence of neurological abnormalities in IUGR infants. As can be seen, the reliability of S100B values as a diagnostic test was greatest from the first urination and with a cut-off >1.0 µg/L, the value at which S100B achieved a sensitivity of 100% (CI_5–95%_: 73.4–100) and a specificity of 100% (CI_5–95%_: 96.9–100) as a single marker for predicting the occurrence of neonatal death (area under the ROC curve: 1.000; CI_5–95%_: 0.972–1.0) (Table 4 and Figure 1). The predictive values of S100B levels at the remaining time-points, at cut-offs >1.0 µg/L, gave similar results (Table 4).

**Table 4:** Sensitivity, specificity and predictive values at different monitoring time-points of serial urinary S100B levels as diagnostic test for early neonatal death detection, at cut-off (>1 µL) identified by the ROC curve analysis.

Monitoring	PPV (%)	NPV (%)	Sens (%)	Spec (%)	AUC
Time-points	(CI_5–95%_)	(CI_5–95%_)	(CI_5–95%_)	(CI_5–95%_)	(CI_5–95%_)
First urination	100.0	100.0	100	100	1.0
	(98–100)	(98–100)	(73.4–100.0)	(96.9–100.0)	(0.972–1.000)
24 hours	100.0	100.0	100	100	1.0
	(98–100)	(98–100)	(73.4–100.0)	(96.9–100.0)	(0.972–1.000)
48 hours	100.0	100.0	100	100	1.0
	(98–100)	(98–100)	(73.4–100.0)	(96.9–100.0)	(0.972–1.000)
96 hours	100.0	100.0	100	100	1.0
	(98–100)	(98–100)	(73.4–100.0)	(96.9–100.0)	(0.972–1.000)

Abbreviations: **PPV,** positive predictive value; **NPV,** negative predictive value; **Sens,** sensitivity; **Spec,** specificity; **AUC,** area under the curve; **CI_5–95%_**, lower and upper 95% Confidence Interval.

In contrast, SNAP-PE scores at the cut-off of 38 combined a sensitivity of 50% (CI_5–95%_: 21.2%–78.8%) with a specificity of 88.2% (CI_5–95%_: 81.0%–93.4%) for predicting brain damage (area under the ROC curve: 0.703; CI_5–95%_: 0.617–0.780) (Figure 1). The SNAP-PE AUC was significantly (P = 0.001) lower than that calculated for S100B at first urination (SD: 0.0874; difference between areas: 0.297; CI_5–95%_: 0.125–0.468) (data not shown).

Twelve of 132 patients died with 7 days of birth,giving an overall neonatal death rate in our population of 9.1% (CI_5–95%_: 4.17%–14.02%). This was the predicted probability of ominous outcome prior to measuring S100B (*pre-test probability*). With regard to predicting neonatal death, when only S100B was available the positive predictive value was 100.0% (CI_5–95%_: 98%–100%) and the negative predictive value 100.0% (CI_5–95%_: 98%–100%). When only SNAP-PE computation was available, its positive and negative predictive values were 30.0% (CI_5–95%_: 9.9%–20.1%) and 5.71% (CI_5–95%_: 1.4%–10.0%), respectively.

## Discussion

The present study provides evidence that early after a severe hypoxic insult there is an increase in urine concentrations of S100B, a brain constituent, and that such an increase only occurs in asphyxiated infants who will have a poor neonatal outcome, i.e. neonatal death within 7 postnatal days. The results in healthy controls and in infants suffering hypoxic ischemic encephalopathy but not ominous outcome fit with previous observations [15], [16].

The data relating to asphyxiated newborns with ominous outcome are consistent with previous observations, in which the presence of elevated S100B protein concentrations in biological fluids (amniotic fluid, urine and blood) correlated with: i) the occurrence of unexplained fetal death in the third trimester of pregnancy [13]; ii) the occurrence of an ominous outcome in the first week of age, in preterm infants [14]; iii) the possibility to evaluate cerebral death in adult patients when S100B measurement is included among peri-mortem procedures [17].

The fact that S100B is known to be essentially absent from the kidney [18], and the evidence that early and severe damage to the central nervous system is reasonably held to be responsible for a continuous release of S100B protein into the systemic circulation and, finally, into the urine [3], [14], together support the concept that: i) the notably high concentrations of the protein detected in urine originate in the nervous system; ii) the release of S100B constitutes a warning sign of severe brain insults, and; iii) a higher secretion of S100B correlates with more severe brain injury. Indeed, the same pattern of increased S100B in urine has been shown in infants suffering perinatal complications such as acute and chronic hypoxia [15], [16], [19], cerebral bleeding [3], [10], [11], or during risky procedures for life-threatening conditions (extracorporeal membrane oxygenation, cardiopulmonary by-pass) [20], [21].

S100B may be released, at least in part, from other sites in which it is concentrated, such as adipose tissue and placenta [12], [22], [23]. However, data on the presence of the protein in adipocytes are currently inconclusive, while a placental origin can be reasonably excluded, bearing in mind the limited half-life of the protein (about 1hour) [24].

In the present study we also demonstrated the possibility of identifying newborns at higher risk of early neonatal death in the first hours after birth. Indeed, measuring S100B in the urine of asphyxiated newborns at a stage when standard diagnostic procedures are still silent or unreliable yields positive and negative predictive values relating to the early detection of infants at risk of ominous outcome. S100B was the only parameter that correlated with the occurrence of neonatal death (as shown by multiple logistic regression analysis). Moreover, we found that in newborns with urine S100B levels above the thresholds defined by the ROC curve analysis (>1.0 µL) the probability (positive predictive value) of neurological sequelae was as high as 100%, while it was 0% if these levels were below the threshold: these concentrations thus showed positive and negative predictive values that differ from the overall prevalence of neonatal death (10.2%) in the study population. These findings would need to be confirmed in larger similar studies, since the sample size of the present study was fairly small. They nonetheless appear relevant, since neurological examination and other standard monitoring procedures were unable to detect which asphyxiated infants would develop an adverse neurological outcome and which would not. S100B appears to satisfy the criteria required for a marker in perinatal medicine: i) good reproducibility and simple performance of measurements: the immunoluminometric assay for protein quantification is rapid (about 1-h), inexpensive and simple to perform; ii) detection in a wide range of biological fluids, offering the possibility of reducing perinatal stress; iii) possible employment in longitudinal monitoring, thanks to to its half-life; iv) well-established use as an early and quantitative marker of brain lesion. Urine samples are, moreover, much more easily collected than blood, which is usually required for other brain marker procedures, and are thus more convenient, bearing in mind that the need for repeated blood sampling is widely known to be associated with a risk of anemia [25]. The use of urine samples thus benefits the care of high-risk newborns. Factors such as oliguria and renal failure or the effects of sedative drugs that might complicate or hinder the collection of urine samples, especially in the first hours after birth, were not encountered in the present study, partly because of the small amount of urine needed for S100B protein measurements (100 µL).

In conclusion, in the present study we found that asphyxiated newborns who later died had high S100B levels in urine, and that the measurement of this protein offers additional support in clinical practice, as a further parameter able to identify newborns at risk. Indeed, the early increase in S100B urine concentrations, due to an early release of the protein from the brain, may offer a direct indicator of active cell damage at a stage when standard diagnostic procedures are unable to detect patients at risk, and may represent a warning sign of impending neonatal death.

## Materials and Methods

### Participants

Between January 1, 2001 and December 1, 2006 we performed a cross-sectional study with urine collected consecutively at our tertiary referral centers for Neonatal Intensive Care Units (NICUs) from 132 term newborns (37–42 wks of gestation; mean 39±2 wks), of whom 72 (mean±SD gestational age: 39±1 wks) were healthy with no overt neurological syndrome (healthy group) and the remaining 60 suffered perinatal asphyxia (PA).

The healthy group was composed of newborns who developed jaundice and were hospitalized for 96 hrs in order to investigate blood bilirubin levels and blood pH (including ion concentrations) in the postnatal period (i.e. 24, 48 and 96 hrs ). Newborns who suffered PA were subsequently classified, according to their outcome at 7 days, either as asphyxiated infants complicated by hypoxic ischemic encephalopathy (HIE) with no ominous outcome (HIE Group, n = 48), and infants who died within the first week after birth (Ominous Outcome Group; n = 12; mean±SD gestational age: 39±2 wks). Informed written consent was obtained from all parents of patients prior to inclusion in the study, for which local (Gaslini Children's Hospital University of Genoa and Department of Neonatology, Cairo University) Human Investigations Committee approval was obtained.

All asphyxiated newborns were delivered by emergency cesarean section due to acute fetal distress, defined according to the American College of Obstetricians and Gynecologists as non-reassuring fetal status (bradycardia, late deceleration of the fetal heart rate, severe and repetitive variable deceleration of the fetal heart rate, reduced beat-to-beat variability). Asphyxia was defined according to an Apgar score of <3 at the 5^th^ minute, pH <7.0, or BE<−12 in cord blood or venous blood taken from newborns within 60 min after birth, or the need for positive pressure ventilation (>3 minutes) [26]. Infants who met 3 or more of the above clinical and biochemical parameters were included in the asphyxia group. All patients had a normal karyotype and were free of detectable anomalies; fetuses with malformations or congenital heart disease and those born to women exposed to alcohol or tobacco smoke were excluded from the study. Other exclusion criteria were congenital or perinatal infections including chorioamnionitis, intrauterine growth retardation, multiple pregnancies, maternal drug addiction.

On admission to the NICU we routinely assessed the clinical parameters of all newborns [red blood cell count (RBC), venous blood pH, ion concentrations, plasma glucose levels], and performed a daily neurological examination [27].

S100B protein levels were measured in urine at first urination (time 0) and 24 (time 1), 48 (time 2) and 96 (time 3) hrs after birth. The first urination occurred within the first 6 hours after birth (mean 3 hours). In this regard, the healthy group included only infants in whom spontaneous urination occurred at least four times during the collection times.

The results were correlated with the occurrence or not of ominous outcome within 7 days after birth. Cerebral ultrasound and neurological patterns were assessed at the same time as urine sampling by a single examiner in each Center who did not know the results of the urine test. Finally, the severity of illness in the first 24 hrs after birth was measured using the Score for Neonatal Acute Physiology-Perinatal Extension (SNAP-PE) [28].

### Cranial Assessment

Standard cerebral ultrasonography was performed by a real-time ultrasound machine (Acuson 128SP5, Mountain View CA, USA) using a transducer frequency emission of 3.5 MHz.

### Neurodevelopmental outcome

In the asphyxiated group, the presence within the first 7 days after birth of HIE was classified according to the criteria described by Sarnat and Sarnat [29]. HIE was defined as mild if hyperexcitability or hypotonia persisted without seizures for at least 72 hours after birth; as moderate if the infant was lethargic and had hypotonia, weak primitive reflexes, and seizures; and as severe if the infant showed frequent seizures, apnea, flaccid weakness, or coma. EEG traces were recorded in the asphyxiated infants within the first 5 days from birth.

Neurological examination was performed at the same time-points as urine sampling. Neonatal neurological conditions were classified using a qualitative approach as described by Prechtl [27], assigning each infant to one of three diagnostic groups: normal, suspect or abnormal. An infant was considered to be abnormal when one or more of the following neurological syndromes was present: hyper- or hypokinesia, hyper- or hypotonia, hemisyndrome, apathy syndrome, hyperexcitability syndrome. An infant was classified as suspect if only isolated symptoms were present but no defined syndrome.

### S100B measurement

Urine samples were collected at each time-point, immediately centrifuged at 900 *g* for 10 min, and stored at −70°C. We measured S100B in urine using a commercially available immunoluminometric assay (Lia-mat Sangtec 100, AB Sangtec Medical, Bromma, Sweden), and measurements were performed in duplicate according to the manufacturer's instructions. This assay is specific for the β subunit of the S100 protein and measures the β subunit using three MAb (SMST 12, SMSK 25 and SMSK 28). The β subunit of the S100 protein is known to be predominant (80–96%) in the human brain [30], [31]. The assay detection limit was 0.02 µg/L: precision was less than 5.5% for intra-assay and less than 10.1% for inter-assay.

### Statistical analysis

The Kolmogorov-Smirnov test was used to evaluate whether the distribution of data was Gaussian. Comparison between proportions was performed using Fisher's exact test. Comparison of fetal and neonatal monitoring parameters between groups was performed by the Mann-Whitney U test, Kruskal-Wallis one way ANOVA followed by *post-hoc* Dunn's test when the data were not normally distributed. Changes in S100B levels [median, lower and upper 95% Coefficient Intervals (CI)] in urine at the different monitoring time-points were evaluated using the repeated-measures ANOVA test followed by post-hoc Tukey's Multiple Comparison Test, and Friedman's test followed by post-hoc Dunn's Multiple Comparison Test when the data were not normally distributed. Differences between groups were tested using the Mann-Whitney U test.

Multiple forward stepwise regression analysis was performed with S100B as the dependent variable to analyze the influence of various clinical parameters [renal function, gender, gestational age, maternal antenatal steroid treatment, presence or absence of antenatal infection, normal or abnormal neurological follow-up at one week of age, delivery mode, Apgar scores at 1 and 5 minutes, birthweight, incidence of Respiratory Distress Syndrome (RDS)] on the occurrence of neonatal death.

We used the cut-off indicated by Receiver Operating Characteristic (ROC) analysis [32], [33] to evaluate the positive and negative predictive values, specificity and sensitivity, and likelihood ratios with their respective 95% confidence bounds. Thus, we estimated the probability of neonatal death in asphyxiated newborns and compared it with the pretest probability, defined as the prevalence of neonatal death in the whole group of patients [34].

Statistical analysis was performed using the GraphPad Prism version 3.00 for Windows (GraphPad Software, Inc., San Diego, CA). A value of P<0.05 was considered significant.
